# Cost-Effectiveness of Lung Cancer Screening Using Low-Dose Computed Tomography Based on Start Age and Interval in China: Modeling Study

**DOI:** 10.2196/36425

**Published:** 2022-07-06

**Authors:** Zixuan Zhao, Lingbin Du, Yuanyuan Li, Le Wang, Youqing Wang, Yi Yang, Hengjin Dong

**Affiliations:** 1 Department of Science and Education of the Fourth Affiliated Hospital, Center for Health Policy Studies School of Public Health Zhejiang University School of Medicine Hangzhou China; 2 Department of Cancer Prevention Cancer Hospital of the University of Chinese Academy of Sciences, Zhejiang Cancer Hospital Hangzhou China; 3 Department for Science and Education Hangzhou Ninth People’s Hospital Hangzhou China

**Keywords:** cost-effectiveness analysis, low-dose computed tomography, screening, lung cancer, China

## Abstract

**Background:**

Lung cancer is the most commonly diagnosed cancer and the leading cause of cancer-related death in China. The effectiveness of screening for lung cancer has been reported to reduce lung cancer–specific and overall mortality, although the cost-effectiveness, optimal start age, and screening interval remain unclear.

**Objective:**

This study aimed to assess the cost-effectiveness of lung cancer screening among heavy smokers in China by incorporating start age and screening interval.

**Methods:**

A Markov state-transition model was used to assess the cost-effectiveness of a lung cancer screening program in China. The evaluated screening strategies were based on a screening start age of 50-74 years and a screening interval of once or annually. Transition probabilities were obtained from the literature and validated, while cost parameters were derived from databases of local medical insurance bureaus. A societal perspective was adopted. The outputs of the model included costs, quality-adjusted life years (QALYs), and lung cancer–specific mortality, with future costs and outcomes discounted by 5%. A currency exchange rate of 1 CNY=0.1557 USD is applicable. The incremental cost-effectiveness ratio (ICER) was calculated for different screening strategies relative to nonscreening.

**Results:**

The proposed model suggested that screening led to a gain of 0.001-0.042 QALYs per person as compared with the findings in the nonscreening cohort. Meanwhile, one-time and annual screenings were associated with reductions in lung cancer–related mortality of 0.004%-1.171% and 6.189%-15.819%, respectively. The ICER ranged from 119,974.08 to 614,167.75 CNY per QALY gained relative to nonscreening. Using the World Health Organization threshold of 212,676 CNY per QALY gained, annual screening from a start age of 55 years and one-time screening from the age of 65 years can be considered as cost-effective in China. Deterministic and probabilistic sensitivity analyses were conducted.

**Conclusions:**

This economic evaluation revealed that a population-based lung cancer screening program in China for heavy smokers using low-dose computed tomography was cost-effective for annual screening of smokers aged 55-74 years and one-time screening of those aged 65-74 years. Moreover, annual lung cancer screening should be promoted in China to realize the benefits of a guideline-recommended screening program.

## Introduction

Lung cancer is a leading cause of death in China and globally. The incidence of lung cancer has recently increased dramatically, both in urban and rural areas, and it is currently the most common form of cancer in China. According to the National Central Cancer Registry of China, in 2015, the incidence of lung cancer was 57.26 cases/100,000 persons and the associated mortality rate was 45.87 deaths/100,000 persons, accounting for 20% and 27% of the values for all cancers, respectively [1]. At present, about 70%-75% of lung cancer patients are diagnosed in the middle or advanced stage of the disease [2]. Although there has been remarkable progress in treatment, the 5-year survival rate of patients with advanced lung cancer (stage IV) remains poor, at only 4.2% [3]. A previous study reported that surgical resection in the early stage of lung cancer (stage I) could significantly improve the 10-year survival rate to 92% [4]. Moreover, the disease burden of lung cancer in China is expected to substantially increase labor costs and medical expenditure in the near future. Therefore, promoting prevention, early diagnosis, and timely treatment can improve the prognosis and reduce the disease burden of lung cancer in China.

The effectiveness of low-dose computed tomography (LDCT) for the screening of lung cancer has been confirmed by the National Lung Screening Trial conducted at 33 medical centers in the United States [5]; the UK Lung Cancer RCT Pilot Screening Trial [6], a randomized controlled trial of LDCT screening for lung cancer versus usual care; and the Detection of Lung Cancer Through Low-dose CT Screening Trial conducted by the Dutch Cancer Society [7]. Several other studies have been conducted to explore the cost-effectiveness of lung cancer screening, although most were conducted in the United States and Europe. These studies reported notable differences in disease burden and treatment costs as compared with the findings in China. For example, the incremental cost-effectiveness ratio (ICER) has been reported to be €19,302 (US $22,542) per life year gained and €30,291 (US $35,377) per quality-adjusted life year (QALY) gained in Germany [8], while the ICER has been reported to be US $52,000 per life year gained and US $81,000 per QALY gained in the United States [9]. However, there has been only 1 similar study conducted in China, but this was limited to early versus nonearly lung cancer, which could have underestimated screening effectiveness [10]. In addition, risk factors and the epidemiology of lung cancer differ among countries. Therefore, the aim of this study was to evaluate the cost-effectiveness of LDCT for the screening of lung cancer in China from a societal perspective.

## Methods

### Study Design

This study was conducted in 2 steps. In the first step, a Markov state-transition model with a lifetime horizon was used to mimic the natural progression of lung cancer and assess the potential impact of LDCT screening compared with a lack of screening in a Chinese cohort aged 50 to 74 years. In the second step, the Markov state-transition model combined with real-world data was used to estimate the ICER of each specific screening strategy as compared with nonscreening. A discount rate of 5% was applied to the costs of both strategies. Important assumptions in this study are summarized in Textbox 1.

**Summary of key assumptions.**

**Description of assumptions**

- A simulated cohort of heavy smokers at a start age of 50-74 years was assumed to be followed up until the age of 79 years (mean life expectancy in China) or death.
- A heavy smoker in this study was defined as a current smoker who smokes at least 20 pack-years.
- Individuals in the screened cohort were assumed to undergo screening by low-dose computed tomography once or annually, and those with positive screening results were assumed to have undergone diagnostic biopsies.
- While in the maintenance cancerous stages, the maintenance cost by stage was assumed to be 10% of the treatment cost.
- All costs were expressed in CNY (2021; 1 CNY=0.1557 USD).
- Future costs and effectiveness were discounted by 5%.
- Adherence to screening and follow-up was assumed to be 100%.

### Study Population

The model simulated a cohort of 100,000 heavy smokers in China aged 50 to 74 years until the age of 79 years or death. A heavy smoker was defined as a current smoker who smokes at least 20 pack-years according to the China National Lung Cancer Screening Guidelines with LDCT (2018 version) [11] and the Cost-effectiveness Evaluation of the 2021 US Preventive Services Task Force Recommendation for Lung Cancer Screening [12].

### Markov Model and Transition Probabilities

**Figure 1:**
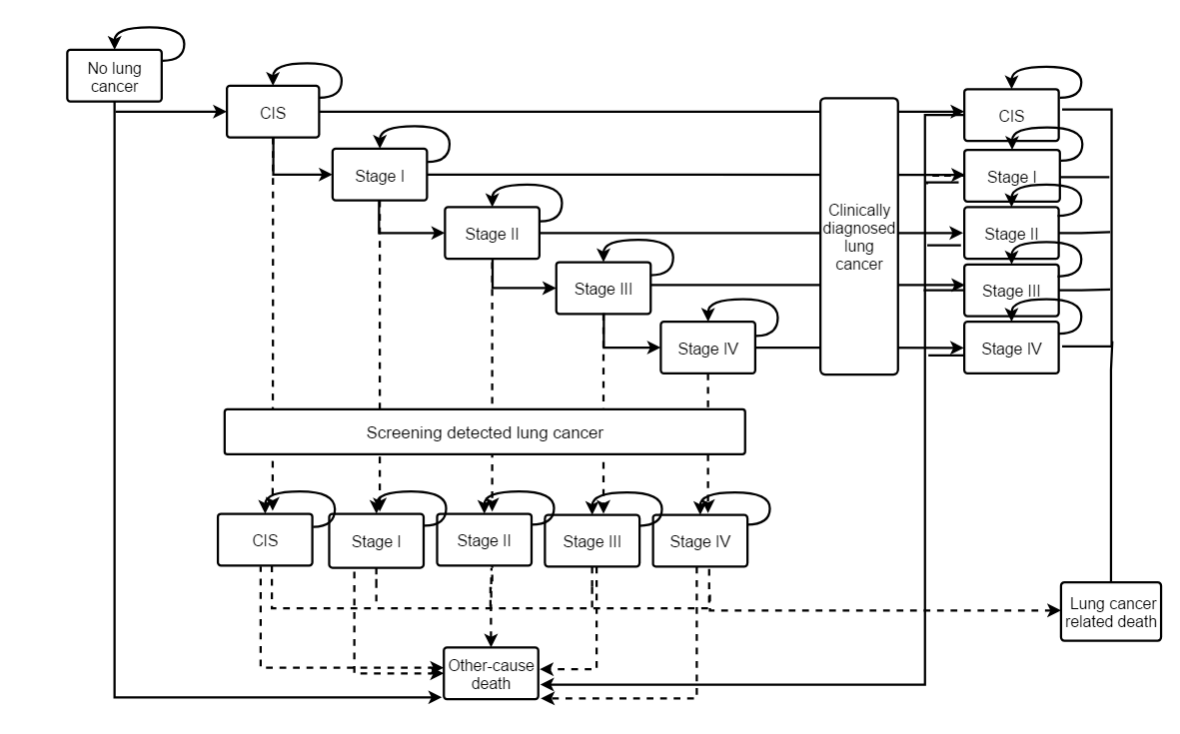
Schematic diagram of natural history for lung cancer screening. CIS: carcinoma in situ.

Lung cancer is assumed to progress sequentially from less advanced to more advanced preclinical stages, as depicted in Figure 1. The following 5 stages are distinguished based on the American Joint Committee on Cancer (AJCC) Cancer Staging Manual, 8th edition: carcinoma in situ (CIS), stage I, stage II, stage III, and stage IV [13]. In this study, stages IA, IB, IIIA, and IIIB were not considered because data were not available for clinical practice in population-based cancer registries in China [14]. The probability of deterioration from a healthy state to all-cause death was retrieved from the 2010 Population Census of the People’s Republic of China [15]. The probability of lung cancer–specific death was retrieved from the published literature [16]. Parameters of disease progression from a healthy state to lung cancer were based on the incidence of lung cancer among smokers in China [17]. The incidence of smokers was modeled as a multiplicative function of smoking rate, age, and sex-specific parameters. The incidence of lung cancer in the general population by sex and age (I_G_) served as the baseline incidence. Specifically, the incidence of lung cancer for smokers (I_S_) was modeled as I_S_ = OR *I_G_ / (1 + (OR – 1)) * R_S_, and the incidence of lung cancer for nonsmokers (I_N_) was modeled as I = I_S_ × R + I_N_ × (1 – R), where OR is the odds ratio for the incidence of lung cancer in smokers, which was extracted from a previous publication [16], R is the proportion of smokers by sex and age reported in the Global Adult Tobacco Survey [18], and I is the incidence of lung cancer in the general population of China. Finally, the incidence of lung cancer (I_20_) among smokers in China was modeled as I_20_ = I_N_ × RR, where the relative risk (RR) of lung cancer (>20 pack-years) attributable to smoking was derived from the published literature [19].

Individuals in the nonscreened cohort were diagnosed based on symptoms. The probability of progression to a more advanced stage of lung cancer or a clinical diagnosis, as described by Ten Haaf et al [20] and a hospital-based multi-center retrospective clinical epidemiological survey in China [21], is detailed in Table 1 [15-19,21-26]. Overall, 19.0% of lung cancer cases were clinically detected in stage I, 16.5% in stage II, 34.7% in stage III, and 29.9% in stage IV.

It was assumed that patients in the screened cohort underwent screening by LDCT at least once or annually and those with positive results underwent additional testing, including biopsy. The positive result rate and proportion of lung cancer by stage were derived from the Wenling lung cancer screening program, which was initiated in 2018 to conduct annual LDCT screening of local high-risk populations over a 3-year period. Of 10,175 asymptomatic individuals who were screened in 2018, 65 (0.64%) were diagnosed with lung cancer (Table 1). Annual screening was conducted in accordance with the protocol of the Cancer Screening Program in Urban China to determine the morphology and size of nodules [22]. The specificity and sensitivity of LDCT for screening of lung cancer were derived from the results of the Multicenter Italian Lung Detection trial [23]. The probability of progression to a more advanced stage or a maintenance state is detailed by stage in Table 1, as described in previous studies [16,22,24]. The proposed model was validated by comparing key outcomes to external empirical data that were not used for model development (Multimedia Appendix 1).

**Table 1:** Input parameters of the Markov model for lung cancer screening.

Variable		Base case value				Distribution		Source
		Male	Female	Overall				
**Lung cancer incidence in the general population (per 100,000 persons) by age in years**								
	50-54	81.0559	89.6626	N/A^a^	Beta		[17]	
	55-59	162.0833	112.4574	N/A	Beta		[17]	
	60-64	256.0943	154.6871	N/A	Beta		[17]	
	65-69	373.6808	190.2521	N/A	Beta		[17]	
	70-74	498.0681	242.6310	N/A	Beta		[17]	
**Smoking rate in the general population**								
	50-64	0.60	0.04	N/A	Beta		[18]	
	65-74	0.45	0.07	N/A	Beta		[18]	
RR^b^ (>20 pack-years)		N/A	N/A	3.87	Beta		[19]	
**Proportion of lung cancer by stage (nonscreened cohort)**								
	CIS^c^	N/A	N/A	0.000	Beta		[21]	
	I	N/A	N/A	0.190	Beta		[21]	
	II	N/A	N/A	0.165	Beta		[21]	
	III	N/A	N/A	0.346	Beta		[21]	
	IV	N/A	N/A	0.299	Beta		[21]	
**Proportion of lung cancer by stage (LDCT^d^ screened cohort)**							Wenling lung cancer screening program	
	CIS	N/A	N/A	0.0370	Beta		N/A	
	I	N/A	N/A	0.6852	Beta		N/A	
	II	N/A	N/A	0.0370	Beta		N/A	
	III	N/A	N/A	0.1852	Beta		N/A	
	IV	N/A	N/A	0.0556	Beta		N/A	
Sensitivity of LDCT (%)		N/A	N/A	79	Beta		[23]	
Specificity of LDCT (%)		N/A	N/A	81	Beta		[23]	
**Mortality of all-cause death (%) by age group**								
	50-54	N/A	N/A	3.59	Beta		[15]	
	55-59	N/A	N/A	4.73	Beta		[15]	
	60-64	N/A	N/A	8.19	Beta		[15]	
	65-69	N/A	N/A	12.99	Beta		[15]	
	70-74	N/A	N/A	21.08	Beta		[15]	
**Lung cancer mortality rate in the general population (per 100,000 persons) by age group**								
	50-54	N/A	N/A	28.81	Beta		[16]	
	55-59	N/A	N/A	52.86	Beta		[16]	
	60-64	N/A	N/A	101.93	Beta		[16]	
	65-69	N/A	N/A	153.34	Beta		[16]	
	70-74	N/A	N/A	248.57	Beta		[16]	
**Transition probabilities (1 year)**								
	Lung cancer stage CIS to I	N/A	N/A	0.0980	Beta		[24]	
	Lung cancer stage I to II	N/A	N/A	0.3682	Beta		[22]	
	Lung cancer stage I to III	N/A	N/A	0.0328	Beta		[22]	
	Lung cancer stage I to IV	N/A	N/A	0.0745	Beta		[22]	
	Lung cancer stage II to III	N/A	N/A	0.2260	Beta		[22]	
	Lung cancer stage II to IV	N/A	N/A	0.1510	Beta		[22]	
	Lung cancer stage III to IV	N/A	N/A	0.1455	Beta		[22]	
	Lung cancer stage CIS to death	N/A	N/A	0.00	Beta		[16]	
	Lung cancer stage I to death	N/A	N/A	0.04	Beta		[16]	
	Lung cancer stage II to death	N/A	N/A	0.07	Beta		[16]	
	Lung cancer stage III to death	N/A	N/A	0.13	Beta		[16]	
	Lung cancer stage IV to death	N/A	N/A	0.18	Beta		[16]	
**Utility by stage**								
	CIS	N/A	N/A	0.87	Beta		[25]	
	I	N/A	N/A	0.84	Beta		[26]	
	II	N/A	N/A	0.84	Beta		[26]	
	III	N/A	N/A	0.87	Beta		[26]	
	IV	N/A	N/A	0.75	Beta		[26]	
**Cost (CNY^e^)**							Survey data	
	Direct screening cost	N/A	N/A	245.86	Gamma		N/A	
	Indirect screening cost	N/A	N/A	23.07	Gamma		N/A	
	Prediagnosis cost	N/A	N/A	628.36	Gamma		N/A	
	Biopsy diagnosis cost	N/A	N/A	1232.44	Gamma		N/A	
**Treatment cost by stage**								
	CIS	N/A	N/A	47,341.85	Gamma		N/A	
	I	N/A	N/A	53,344.51	Gamma		N/A	
	II	N/A	N/A	83,365.95	Gamma		N/A	
	III	N/A	N/A	90,643.18	Gamma		N/A	
	IV	N/A	N/A	116,471.34	Gamma		N/A	

^a^N/A: not applicable.

^b^RR: relative risk.

^c^CIS: carcinoma in situ.

^d^LDCT: low-dose computed tomography.

^e^A currency exchange rate of 1 CNY=0.1557 USD is applicable.

### Cost Data

The total cost of the screening program included direct expenses (ie, public advertising, management of screening invitations, salaries of staff members, and depreciation of screening equipment) and indirect expenses (ie, transportation and wages for missed work). In addition, the cost of diagnostic biopsies for participants with positive results after initial LDCT was considered. Screening-related costs were retrieved from data provided by the Wenling lung cancer screening program. Costs of treatment of lung cancer by stage were derived from a database of local medical insurance bureaus, which included 4947 patients and 107,248 relevant records. The cost of maintenance by stage accounted for 10% of the total treatment cost. All costs in this study are expressed in Chinese yuan (CNY) at a discount of 5% of rates in 2018. A currency exchange rate of 1 CNY=0.1557 USD is applicable.

### Quality of Life

The putative benefit of cancer screening for early diagnosis was assumed to be a difference in life expectancy and QALY after treatment. As the severity and responsiveness to treatment vary according to stage, the specified utility score for each stage was used for calculation [25,27]. The utility score was 0.84 for lung cancer stage I/II, 0.87 for CIS and stage III, and 0.75 for stage Ⅳ (Table 1).

### Evaluation Strategies

As the scheduled screening program included several key characteristics, different combinations of screening intervals and start ages, as well as a nonscreening cohort, were evaluated (Table 2). In order to achieve more realistic economic evaluation outcomes, one-time screening was applied in this study because no periodic screening program has been implemented nationwide in China and most of the study participants were screened for lung cancer only once. Therefore, the rationale of one-time screening was based on limited financial support for lung cancer screening programs in China. Moreover, strategies with annual screening from different start ages were simulated to determine whether efforts are needed to promote periodic screening programs in China in order to realize relative benefits based on current guidelines.

**Table 2:** Evaluation strategies.

Scenario	Screening tool	Screening interval	Start age (years)
LDCT^a^#1	LDCT	Annual	50, 55, 60, 65, and 70
LDCT#2	LDCT	One time	50, 55, 60, 65, and 70
Nonscreening	N/A^b^	N/A	50, 55, 60, 65, and 70

^a^LDCT: low-dose computed tomography.

^b^N/A: not applicable.

### Outcomes and Cost-Effectiveness

The main outcomes of the cost-effectiveness analysis for each strategy were QALYs and total costs. The ICER was calculated by dividing the incremental costs by the incremental QALYs gained for each screening strategy as compared to nonscreening. In China, there is no regulated or published cost-effectiveness threshold. Hence, the threshold recommended by the World Health Organization (WHO) is commonly used. Given that 3 times the gross domestic product per capita was used as a reference point, a tentative threshold value of 212,676 CNY was adopted in this study.

### Sensitivity Analysis

The Markov state-transition model was developed using TreeAge Pro 2021 software (TreeAge Software, Inc). The parameters of direct screening cost, maintenance cost, discount rate, consumer price index (CPI) rate, incidence rate of heavy smokers, and specificity and sensitivity of LDCT uncertainty were investigated by 1-way deterministic sensitivity analyses. The costs of direct screening, as well as maintenance costs, CPI rate, and incidence rate of heavy smokers, were set to vary by 30% as compared to base values. The discount rate was set to range from 0% to 8%, and the sensitivity and specificity of LDCT were set to range from 0.63 to 0.95 and 0.65 to 0.97, respectively. Input parameters were randomly drawn from beta or gamma distributions (Table 1).

## Results

The results of the model suggested that the QALYs of the screening cohort increased by 0.001 to 0.042 as compared to that of the nonscreening cohort. The reduction in lung cancer–associated mortality ranged from 0.004% to 1.171% for one-time screening and from 6.189% to 15.819% for annual screening (Table 3). The average costs per person in the nonscreening cohort, one-time screening cohort, and annual screening cohort were 24,896.93, 25,521.61, and 34,105.70 CNY, respectively, at a start age of 50 years, which seemed to be the most noncost-effective among the 5 age groups. Conversely, the most cost-effective start age was 70 years, with ICERs in the one-time screening and annual screening cohorts of 180,280.19 and 119,974.08 CNY per QALY gained. As compared to the nonscreening cohort, the ICER of the screening cohort, regardless of the screening interval, ranged from 119,974.08 to 614,167.75 CNY per QALY gained. Using the WHO threshold of 212,676 CNY per QALY gained, annual screening at a start age of 55-74 years was determined to be the most cost-effective in China. For one-time screening, the cost-effective start age was 65-74 years.

**Figure 2:**
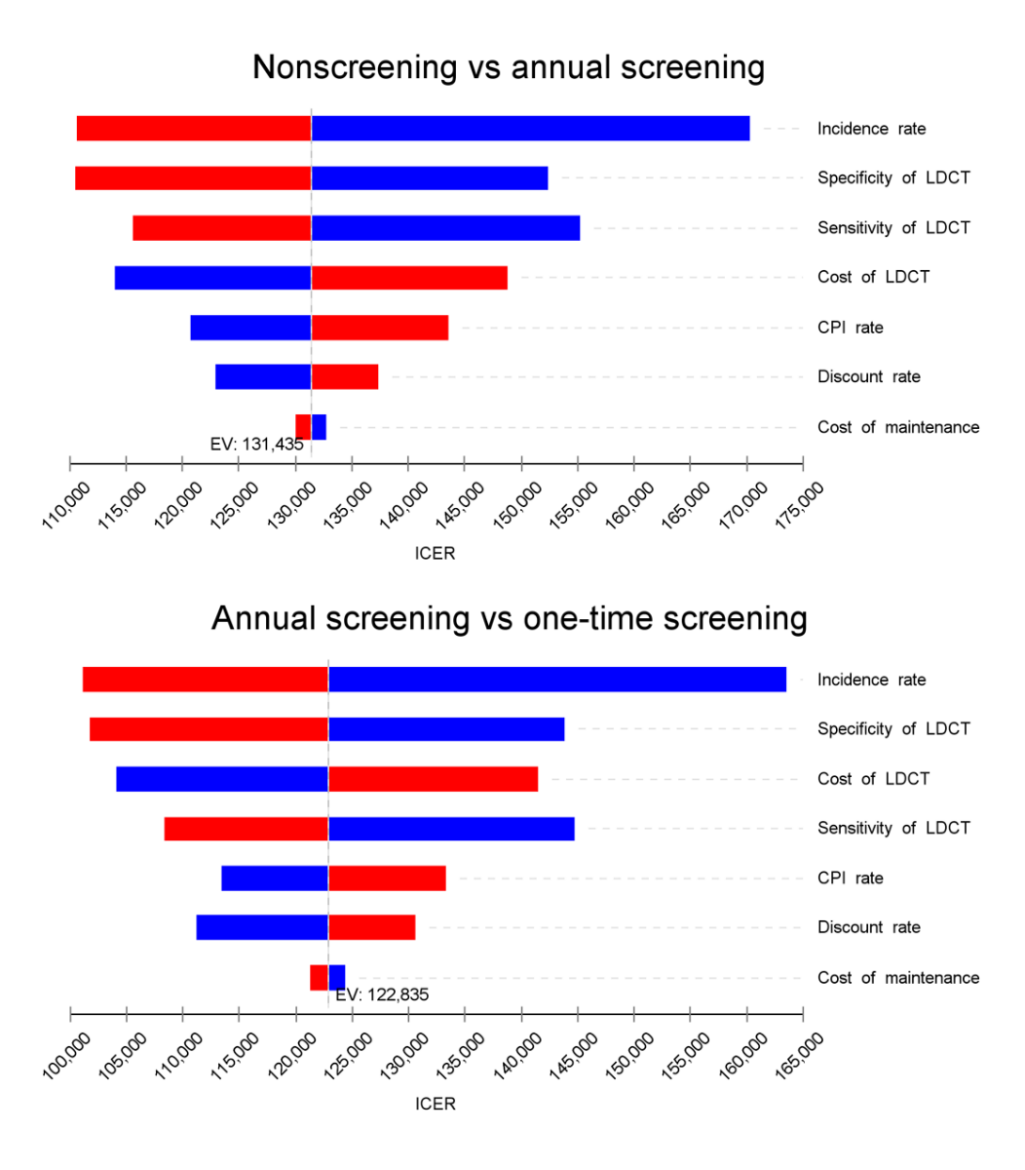
Tornado diagrams. The tornado diagrams illustrate the change in the incremental cost-effectiveness ratio (ICER). The blue column shows the impact of decreasing the input parameters on the results. Similarly, the red column shows the impact of increasing the input parameters on the results. CPI: consumer price index; EV: expected value; LDCT: low-dose computed tomography.

**Figure 3:**
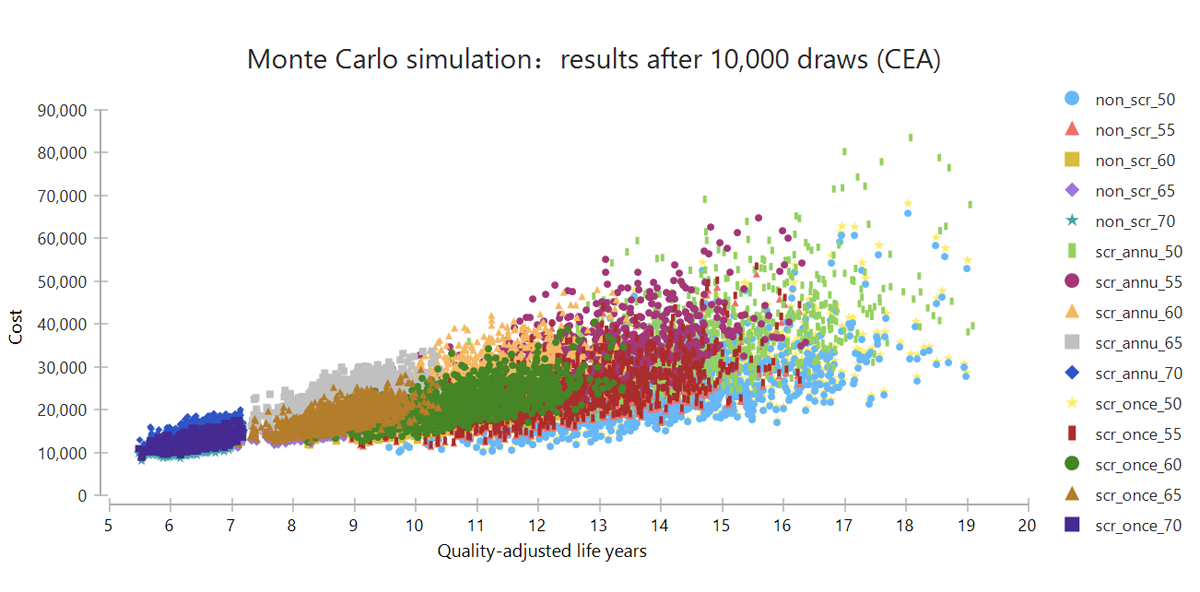
Probabilistic sensitivity analyses. The screening strategies are labeled as follows: screening or not screening interval_start age. CEA: cost-effectiveness analysis; non_scr: nonscreening; scr_annu: annual screening; scr_once: one-time screening.

The sensitivity of the model for the above-mentioned parameters is shown in Figure 2. Generally, the model results were robust with no variation exceeding 212,676 CNY per QALY gained at a start age of 65-74 years. The highest sensitivity was observed for the rate of newly developed lung cancer in heavy smokers. The accuracy parameters of LDCT (ie, sensitivity and specificity) and the direct cost of the screening program had relatively high influences on the ICERs, while variations in discount rates had relatively little influence. After 10,000 repetitions, Monte Carlo simulation revealed that the average ICER ranged from 143,253.62 to 776,678.97 CNY, which was greater than the base ICER (Figure 3).

**Table 3:** Base case results with different screening settings (per 100,000 persons).

Start age and strategy^a^		Cost (CNY,^b^ millions)	QALYs^c^ (10,000 years)	Lung cancer mortality reduction vs nonscreening (%)	ICER^d^ Scr vs Non_scr	ICER Scr_annu vs Scr_once
**50 years**						
	Non_scr	2489.69	135.92	N/A^e^	N/A	N/A
	Scr_once	2552.16	135.93	0.0041	614,167.75	N/A
	Scr_annu	3410.57	136.30	6.1886	245,746.19	235,467.06
**55 years**						
	Non_scr	2380.25	121.21	N/A	N/A	N/A
	Scr_once	2448.97	121.23	0.0145	365,289.96	N/A
	Scr_annu	3176.64	121.62	6.7044	192,119.62	183,886.78
**60 years**						
	Non_scr	2154.69	104.08	N/A	N/A	N/A
	Scr_once	2230.61	104.11	0.0467	263,083.31	N/A
	Scr_annu	2808.69	104.50	7.7816	154,401.89	146,456.38
**65 years**						
	Non_scr	1773.45	84.40	N/A	N/A	N/A
	Scr_once	1860.84	84.45	0.1997	192,574.66	N/A
	Scr_annu	2260.66	84.77	10.0628	131,284.57	122,745.38
**70 years**						
	Non_scr	1184.22	61.56	N/A	N/A	N/A
	Scr_once	1279.79	61.61	1.1705	180,280.19	N/A
	Scr_annu	1476.25	61.80	15.8193	119,974.08	103,182.45

^a^Non_scr: nonscreening; Scr_once: one-time screening; Scr_annu: annual screening.

^b^A currency exchange rate of 1 CNY=0.1557 USD is applicable.

^c^QALY: quality-adjusted life year.

^d^ICER: incremental cost-effectiveness ratio.

^e^N/A: not applicable.

## Discussion

### Principal Findings

This is the first cost-effectiveness analysis of a lung cancer screening program with different start ages and screening intervals using real-world data in China. In summary, using a lifetime societal perspective for one-time or annual LDCT for screening of heavy smokers, the annual screening strategy with a start age of 55-74 years showed strong dominance as compared with the nonscreening strategy. These results were sensitive to the rate of newly developed lung cancer and the specificity of LDCT. As compared with the nonscreening strategy, the one-time screening strategy was cost-effective for patients aged 65-74 years, using a cost-effectiveness threshold of 212,676 CNY per QALY gained. This finding is consistent with that in the UK Lung Screen trial, which demonstrated a long-term benefit from a single screen and provided potentially important data for inclusion in future modeling studies to optimize the screening interval [26]. All simulated results of probabilistic sensitivity analysis were robust when the main input parameters were varied.

Although the analytical approach was somewhat similar to that in a previous study by Yuan et al [28], the strategies were enriched by adding screening intervals and thus arrived at different conclusions. First, Yuan et al predicted that the ICERs of all screening strategies with a start age of 40-74 years were 3-fold lower than the gross domestic product per capita. However, this result is consistent with only part of the strategies in this study. Second, Yuan et al predicted a minimum ICER at a start age of 65 years, whereas the results of this study demonstrated a decreasing trend in ICER per QALY gained from a start age of 50-74 years, regardless of the screening interval. These differences may have resulted from a combination of several factors. For example, Yuan et al used a discount rate of 3%, while a rate of 5% was adopted in this study in accordance with the China Guidelines for Pharmacoeconomic Evaluations [29], and staging was simplified in the Markov model by ignoring the CIS stage. For comparison between annual screening and nonscreening, the ICER of 119,974 to 245,746 CNY in this study is comparable to previous estimates of US $24,934, US $49,200-96,700, and US $33,825 per QALY gained reported by studies conducted in New Zealand, the United States, and Canada, respectively [30-32].

Regarding the implications of policies related to lung cancer screening, the China National Lung Cancer Screening Guidelines with LDCT (2018 version) [11] were partially confirmed by the recommendations for lung cancer screening and early diagnosis and treatment guidelines in China [33] from a health economic perspective. Though the updated recommendations for lung cancer screening and early diagnosis and treatment guidelines raised the minimum cumulative smoking exposure from 20 to 30 pack-years relative to the 2018 version, the results were robust according to deterministic 1-way sensitivity analysis. In addition to the low utilization of lung cancer screening programs in China, there is a need to improve the accessibility and affordability of population-based screening programs to better capture the full extent of benefits associated with lung cancer screening. Annual lung cancer screening for heavy smokers at a start age of 55-74 years is considered cost-effective in China. Although screening from the age of 70 years had the lowest ICER per QALY gained as compared to nonscreening, it is unreasonable to simply use a start age of 70 years. An older start age is associated with fewer QALYs obtained.

### Limitations

There were several limitations to this study that should be addressed. First, like most mathematical models, the model used in this study to estimate the incidence of lung cancer in heavy smokers was a simplification of the biological complexity of lung carcinogenesis and neglected the influence of various endogenous and exogenous risk factors, such as family history and residential/occupational exposure to radon, which may have led to underestimation of the incidence of lung cancer in the targeted population. Further, as heavy smokers are more likely to die from other diseases (eg, cardiovascular diseases and other cancers), its application to estimate the general probability of all-cause death in this population might have slightly underestimated the mortality rate in this work. Nevertheless, the use of this nomothetic approach has aided the development of prevention and control strategies against lung cancer in the United States [34]. Second, the cumulative burden of radiation from annual screening with LDCT was not considered. Albert et al reported that annual LDCT would result in additional radiation exposure of 1.5 mSv per year [35]. Still, recent studies have reported that the potential benefit of lung cancer screening to prevent death was greater than the potential harm of increased radiation exposure [36,37]. Third, smoking cessation events and other health-related behavioral changes due to screening participation were not incorporated in the model due to the lack of relevant data. Further research may benefit from the incorporation of patient-level data extracted from on-going randomized controlled trials with microsimulation models for cost-effectiveness analysis of lung cancer screening.

### Conclusion

This economic evaluation revealed that a population-based lung cancer screening program in China for heavy smokers using LDCT could result in more QALYs, although with greater expense than nonscreening. Using the WHO threshold for cost-effectiveness analysis, the annual screening strategy from 55 to 74 years and one-time screening strategy from 65 to 74 years can be considered cost-effective. Moreover, annual screening was the most promising; thus, annual screening should be promoted in China to realize actual benefits.

## Appendix

Multimedia Appendix 1 Validation of the natural history model of lung cancer.

## Abbreviations

CIS: carcinoma in situ
CPI: consumer price index
ICER: incremental cost-effectiveness ratio
LDCT: low-dose computed tomography
OR: odds ratio
QALY: quality-adjusted life year
RR: relative risk
WHO: World Health Organization
