# Correction: Cost-Effectiveness of Lung Cancer Screening Using Low-Dose Computed Tomography Based on Start Age and Interval in China: Modeling Study

**DOI:** 10.2196/43025

**Published:** 2022-10-17

**Authors:** Zixuan Zhao, Lingbin Du, Yuanyuan Li, Le Wang, Youqing Wang, Yi Yang, Hengjin Dong

**Affiliations:** 1 Department of Science and Education of the Fourth Affiliated Hospital, Center for Health Policy Studies School of Public Health Zhejiang University School of Medicine Hangzhou China; 2 Department of Cancer Prevention Cancer Hospital of the University of Chinese Academy of Sciences, Zhejiang Cancer Hospital Hangzhou China; 3 Department for Science and Education Hangzhou Ninth People’s Hospital Hangzhou China

In “Cost-Effectiveness of Lung Cancer Screening Using Low-Dose Computed Tomography Based on Start Age and Interval in China: Modeling Study” (JMIR Public Health Surveill 2022;8(7):e36425), the authors noted an error:

In the originally published article, Affiliations 1 and 4 were inadvertently presented as two different affiliations due to erroneous presentation of organizational units within these affiliations.

In the corrected version, the two affiliations are merged into a single affiliation with the correct order of organizational units. With this update, the numbering and attribution of affiliations to the authors are updated as follows:

Zixuan Zhao^1^; Lingbin Du^2^; Yuanyuan Li^3^; Le Wang^2^; Youqing Wang^2^; Yi Yang^1^; Hengjin Dong^1^

^1^Department of Science and Education of the Fourth Affiliated Hospital, Center for Health Policy Studies, School of Public Health, Zhejiang University School of Medicine, Hangzhou, China

^2^Department of Cancer Prevention, Cancer Hospital of the University of Chinese Academy of Sciences, Zhejiang Cancer Hospital, Hangzhou, China

^3^Department for Science and Education, Hangzhou Ninth People’s Hospital, Hangzhou, China

Affiliation noted in the corresponding author's contact information is also updated accordingly.

The correction will appear in the online version of the paper on the JMIR Publications website on October 17, 2022, together with the publication of this correction notice. Because this was made after submission to PubMed, PubMed Central, and other full-text repositories, the corrected article has also been resubmitted to those repositories.

