# Supplementary material for: Cost-Effectiveness of Lung Cancer Screening Using Low-Dose Computed Tomography Based on Start Age and Interval in China: Modeling Study
Source: JMIR Public Health Surveill. 2022 Jul 6;8(7):e36425. doi: 10.2196/36425 (PMC9301557; doi:10.2196/36425)
Supplement: Multimedia Appendix 1 [file publichealth_v8i7e36425_app1.docx]

Multimedia Appendix 1

- Operational validation for the natural history model of lung cancer

The natural history model of lung cancer simulates the occurrence and development process of cancer in the real world, but in fact, the occurrence and development process of cancer is very complicated, and the accurate natural history process is often difficult to be fully understood. Therefore, there are many conjectures and assumptions about the natural history process when building the model, and the output results of the model have uncertainties. The evaluation of the operational validity of the natural history model thus is of great significance.

1. Parameters in the natural history model

In each cycle, the probability of metastasis from healthy to different stages of lung cancer was the proportion of incidence rate and corresponding proportion. According to a national multicenter lung cancer epidemiological survey, the constituent ratios of carcinoma in situ and invasive carcinoma were 0, 0.190, 0.165, 0.346, 0.299[1] respectively. The incidence rate parameters are from the China cancer registry annual report [2] in 2018. The mortality rate of lung cancer is from the survival analysis of Shanghai population [3]. Since the prognosis of lung cancer in situ is better after surgery, the mortality rate of CIS is set to 0. The mortality of lung cancer removal is obtained by subtracting the age-specific lung cancer mortality from the age-specific all-cause mortality published in the national demographic annual report [2,4]. The initial probability parameters are shown in table 1. In CIS and stage I invasive carcinoma will progressing to the next stage in a row. In addition to, the probability of metastasis is derived from a German lung cancer screening model and adjusted for [5] according to the incidence rate in China and Germany. According to a global review, the 1-year metastasis probability of carcinoma in situ developing to stage I lung cancer is estimated to be 0.098 [6], and the specific metastasis probability parameters are shown in table 2.

Table 1 Initial and death probability of natural history model

| Age | Incidence | Mortality | | | | All-cause mortality(except lung cancer) |
| --- | --- | --- | --- | --- | --- | --- |
|  |  | Stage I | Stage II | Stage III | Stage IV |  |
| 0-5 | 0 | 0.02 | 0.03 | 0.06 | 0.07 | 0.001289 |
| 6-9 | 0 | 0.02 | 0.03 | 0.06 | 0.07 | 0.000299 |
| 10-14 | 0 | 0.02 | 0.03 | 0.06 | 0.07 | 0.000299 |
| 15-19 | 3.2*10^(-6) | 0.02 | 0.03 | 0.06 | 0.07 | 0.000388 |
| 20-24 | 1.29*10^(-5) | 0.02 | 0.03 | 0.06 | 0.07 | 0.000498 |
| 25-29 | 3.5*10^(-5) | 0.02 | 0.03 | 0.06 | 0.07 | 0.000607 |
| 30-34 | 7.34*10^(-5) | 0.02 | 0.03 | 0.06 | 0.07 | 0.000807 |
| 35-39 | 0.000145 | 0.03 | 0.05 | 0.1 | 0.13 | 0.001148 |
| 40-44 | 0.000256 | 0.03 | 0.05 | 0.1 | 0.13 | 0.001712 |
| 45-49 | 0.000466 | 0.04 | 0.07 | 0.1 | 0.18 | 0.002496 |
| 50-54 | 0.000853 | 0.04 | 0.07 | 0.13 | 0.18 | 0.003884 |
| 55-59 | 0.001377 | 0.04 | 0.07 | 0.13 | 0.18 | 0.005645 |
| 60-64 | 0.002056 | 0.04 | 0.07 | 0.13 | 0.18 | 0.009247 |
| 65-69 | 0.002811 | 0.05 | 0.09 | 0.15 | 0.24 | 0.015554 |
| 70-74 | 0.003667 | 0.05 | 0.09 | 0.15 | 0.24 | 0.027761 |
| 75-79 | 0.004058 | 0.06 | 0.13 | 0.27 | 0.35 | 0.044858 |
| 80-84 | 0.003883 | 0.06 | 0.13 | 0.27 | 0.35 | 0.077317 |
| Source | [2] | [3] | [3] | [3] | [3] | [4] |

Table 2 Transition probabilities in natural history model

| Start state | Terminal state | Transition probabilities | Source |
| --- | --- | --- | --- |
| CIS | I | 0.0980 | [6] |
| I | II | 0.3682 | [5] |
| I | III | 0.0328 | [5] |
| I | IV | 0.0745 | [5] |
| II | III | 0.2260 | [5] |
| II | IV | 0.1510 | [5] |
| III | IV | 0.1455 | [5] |
| CIS | dead | 0 | [5] |
| I | dead | 0.04 | [5] |
| II | dead | 0.07 | [5] |
| III | dead | 0.13 | [5] |
| IV | dead | 0.18 | [3] |

1. Data source of validity evaluation indicators

This work intends to build a natural history model of lung cancer suitable for Chinese population. Therefore, when determining the target parameters of model debugging, priority should be given to the publicly reported data of population-specific epidemiology in China, supplemented by the data reported in a single literature when the data are not available. The commissioning objectives include lung cancer incidence (mortality) rate, age standardized incidence (mortality) rate, cumulative incidence (mortality) rate, life expectancy, proportion of different clinical stages, etc. see table 1 for specific indicators and their sources.

The parameters related to morbidity and mortality are derived from the relevant data of annual report for cancer registration during 2018-2020 [7-9]. Since 2008, China has initiated a national cancer registration project and gradually carried out population-based information collection on cancer incidence, death and survival in 31 provinces (municipalities directly under the central government and autonomous regions). Among them, the 2018 annual cancer registration report included data from 501 cancer registries across the country, covering a population of 388 million.

The proportion of different clinical stages of lung cancer in natural state comes from a national multicenter clinical epidemiological survey of lung cancer. The survey systematically reviewed the clinical data of 7184 patients with lung cancer from 2005 to 2014, including the composition ratio of clinical stages [10].

Table 3 Validity indicators and sources

| Indicator | Time range | Data source | |
| --- | --- | --- | --- |
| Incidence（Mortality）crude rate | 2018-2020 | | China Cancer Registration Annual Report |
| Age-standardized incidence（mortality）rate | 2018-2020 | |  |
| Accumulated incidence（mortality）rate | 2018-2020 | |  |
| Proportion of lung cancer | 2005-2014 | | A multicenter clinical epidemiological survey |
| Life expectancy | 2016-2018 | | China Health Statistics Yearbook |
| Age-specific life expectancy | 2017-2019 | | Global Burden of Disease Study（GBD） |

（2）Calculation method for validity indicators

①Lung cancer incidence and mortality

The incidence (mortality) rate, is so-called the crude incidence (mortality) rate, is the ratio of the number of newly diagnosed cases (deaths) of lung cancer to the corresponding population.

Incidence（mortality）rate per 100 000=$\frac{new cases\left（ new cancer deaths \right）}{population at the same period}*100000$

Since the crude incidence (mortality) rate is greatly affected by the age structure of the population, in order to ensure the comparability between the model output results and the cancer registration data, it is necessary to calculate the age standardized incidence (mortality) rate according to the age structure of a standard population. This work uses the population composition of the fifth national census in 2000 and Segi's world standard population to calculate the China standard rate and world standard rate respectively. The calculation formula is:

Age-standardization incidence(mortality) rate per 100000=$\frac{\sum standard population in corresponding age group*age-specific rate}{\sum standard population}$

In addition to the age standardized rate, the cumulative incidence (mortality) rate can also eliminate the influence of age structure and can be used for the comparison of different incidence (mortality) data. Specifically, it refers to the total indicator of cumulative incidence (mortality) rate according to age in a certain age stage. This work selects the cumulative incidence (mortality) rate aged 0-74, the calculation formula is:

Cumulative incidence (mortality) rate（%）=（$\sum（age-specific incidence\left（ \mathrm{mortality} \right）rate*age group））*100$

Table 4 Standard population

| Age group（years） | China Standard population (2000) | Segi’s population |
| --- | --- | --- |
| 0~ | 13793799 | 2400 |
| 1~ | 55184575 | 9600 |
| 5~ | 90152587 | 10000 |
| 10~ | 125396633 | 9000 |
| 15~ | 103031165 | 9000 |
| 20~ | 94573174 | 8000 |
| 25~ | 117602265 | 8000 |
| 30~ | 127314298 | 6000 |
| 35~ | 109147295 | 6000 |
| 40~ | 81242945 | 6000 |
| 45~ | 85521045 | 6000 |
| 50~ | 63304200 | 5000 |
| 55~ | 46370375 | 4000 |
| 60~ | 41703848 | 4000 |
| 65~ | 34780460 | 3000 |
| 70~ | 25574149 | 2000 |
| 75~ | 15928330 | 1000 |
| 80~ | 7989158 | 500 |
| 85+ | 4001925 | 500 |
| Total | 1242612226 | 100000 |

② Proportion for clinical stages

The proportion for clinical stages indicates how much the number of stage-specific new cancer cases accounts for the number of all the new lung cancer cases. The formula is:

Proportion for clinical stages(%)=$\frac{No. of cases of a particular stage}{No. of cases of all stages}$

③ Life expectancy

Life expectancy refers to taking the age-specific mortality rate of a certain year as a fixed value, assuming that it does not change with time, and estimating the average time that people born in a certain period can survive, usually in "years". This work simulates a group of 0-year-old birth cohort, and plans to use the life table method to calculate the life expectancy. The specific calculation formula is as follows:

Age-specific mortality $m_{x}=\frac{Number of deaths during the year}{Average annual population}$

Age-specific dead probability $q_{x}=\frac{2nm_{x}}{2+nm_{x}}$ ，n represents years

Age-specific survival probability $p_{x}={1-q}_{x}$

Relations between age-specific survive population$l_{x}$ and dead population $d_{x}$

$$d_{x}=l_{x}q_{x}$$

$$l_{x+n}=l_{x}-d_{x}$$

Relations between life years $L_{x}$ and total life years $T_{x}$

$$L_{X}=n\frac{l_{x}+l_{x+n}}{2}$$

$$T_{x}=\sum L_{x}$$

1. Validity evaluation of natural history model

① Incidence and mortality

Using the natural history model constructed above to simulate a birth cohort of 1000 people, the results show that the crude incidence and mortality rates of lung cancer are 64.17/100000 and 55.83/100000 respectively, which are similar to the data of China annual cancer registration reports from 2018 to 2020; After standardization by China's population composition, the rate is 37.2/100000, and the rate after the standardization of Segi's world population composition is 36.98/100000, which is basically consistent with the data of 2018-2020 National Cancer Registration annual report; The cumulative incidence rate of 0-74 years by model simulation is basically consistent with the annual data of tumor registration. Compared with the annual data of national cancer registration, the simulation value of mortality is a little bit higher. See table 3 for details.

Table 5 Validity indicator：incidence and mortality of lung cancer

| Indicator | Simulated value | Observed value(Cancer registration report） | | |
| --- | --- | --- | --- | --- |
|  |  | 2015 | 2016 | 2017 |
| Incidence |  |  |  |  |
| Crude rate /100000 | 64.17 | 58.91 | 60.04 | 74.87 |
| ASR China /100000 | 37.20 | 35.57 | 36.04 | 39.23 |
| ASR World /100000 | 36.98 | 35.54 | 36.02 | 47.58 |
| 0-74 cumulative rate(%) | 4.73 | 4.34 | 4.41 | 5.88 |
| Mortality |  |  |  |  |
| Crude rate /100000 | 55.83 | 47.79 | 48.42 | 27.85 |
| ASR China /100000 | 28.78 | 27.99 | 27.95 | 26.24 |
| ASR World /100000 | 28.58 | 27.85 | 27.87 | 26.10 |
| 0-74 cumulative rate(%) | 3.64 | 3.27 | 3.27 | 3.01 |

② Proportion for clinical stages

Figure 1 Proportion for clinical stages

③ Life expectancy

The model simulation reveals that the life expectancy of China's birth cohort is 76.8 years, which is consistent with the observed values (76.5, 76.7 and 77.0) reported from the 2016-2018 China Health Statistical Yearbook [11], and is consistent with the observed value in GBD for 2018, which is 77.4 (95% uncertainty interval: 76.2-78.8) years. The trend of life expectancy corresponding to different ages is basically consistent with the observed value of GBD.

Figure 2 Comparison between GBD observed value and simulation value in life expectancy

**References**

[1] Shi JF, Wang L, Wu N, et al, Clinical characteristics and medical service utilization of lung cancer in China, 2005–2014: Overall design and results from a multicenter retrospective epidemiologic survey, Lung Cancer,2019(128)91-100, doi:10.1016/j.lungcan.2018.11.031.

[2] He, J. and W. Chen, CHINA CANCER REGISTRY ANNUAL REPORT (2018). 2019: People's Medical Publishing House.ISBN:978-7-117-28585-8.

[3] Zhang, M., et al., Survival analysis of patients with lung cancer in Shanghai. China Oncology, 2017.

[4] Tabulation on the 2010 Population Census of the People's Republic of China. 2010, Department of Population and Employment Statistics of the National Bureau of Statistics of China: Beijing, China.

[5] Hofer F, Kauczor HU, Stargardt T. Cost-utility analysis of a potential lung cancer screening program for a high-risk population in Germany: A modelling approach. Lung Cancer. 2018 Oct;124:189-198.doi:10.1016/j.lungcan.2018.07.036.Epub 2018 Jul 24. https://doi.org/10.1016/j.lungcan.2018.07.036

[6] Anindo K. Banerjee. Preinvasive Lesions of the Bronchus,Journal of Thoracic Oncology, 2009.4(4),Pages 545-551.doi:10.1097/JTO.0b013e31819667bd.

[7] National Cancer Institute. 2018 China cancer registry annual report.2018. People's Medical Publishing House, Beijing, China.

[8] National Cancer Institute. 2019 China cancer registry annual report.2019. People's Medical Publishing House, Beijing, China.

[9] National Cancer Institute. 2020 China cancer registry annual report.2020. People's Medical Publishing House, Beijing, China.

[10] Shi JF, Wang L, Wu N, et al, Clinical characteristics and medical service utilization of lung cancer in China, 2005–2014: Overall design and results from a multicenter retrospective epidemiologic survey, Lung Cancer,2019(128)91-100, doi:10.1016/j.lungcan.2018.11.031.

[11] National Health Commission (2018). 2018 China Health Statistical Yearbook Beijing: China Union Medical College Press, 231-239

[12] Institute for health metrics and evaluation (2019).GBD Compare.2019.2021-12-30.Available at:http://vizhub.healthdata.org/gbd-cpmpare/.
